# Conference Summary

**DOI:** 10.6028/jres.110.077

**Published:** 2005-08-01

**Authors:** Dirk Dubbers

**Affiliations:** University of Heidelberg

The study of particle physics with low energy neutrons has a long history starting in the middle of the past century. During most of the time only a rather small number of researchers worked in this field, at least as compared to any of the high energy particle physics collaborations. For instance, in the mid-eighties, when I joined Institut Laue-Langevin (ILL) for several years, I found there only one ILL scientist, the late Walter Mampe, serving the whole community both from Europe and from overseas which came to work at ILL. Today, we see a large number of powerful young groups who have entered the field on each side of the Atlantic and of the Pacific, and I am honoured to give the summary to this conference. So let me run through the topics of this conference to give, at the end, a tabular summary of the basic scientific questions pursued by our community.

The conference started with a session on what is considered by many particle physicists to be the flagship of the field, namely the search for an electric dipole moment (EDM) of the neutron. This topic is closely linked to the question of why so much matter has survived the Big Bang, and to the question of the origin of time reversal violation. Progress in neutron EDM will mainly come from increases in ultracold neutron (UCN) source strength. As we have heard, there are many projects on new powerful UCN sources, both on very small and very large installations, and it is not clear yet who will win this race. Anyway, when significant progress in statistics will be achieved, as we all expect to take place in the near future, then, as history shows, progress in systematics will follow shortly behind. The recent discovery of a new false-effect linked to Bloch-Siegert shifts in non-uniform fields is a good example for this rule.

We then learned about new ideas on neutron-antineutron oscillations. Recently I was asked what I think of having a new neutron oscillation project. This made me think of a dear colleague, who, many years ago, said in a summary talk to the first of this series of conferences: do not bother with free neutron decay any more, the best people have worked on it, and no one will do better. My advice to younger colleagues: Do not listen too much to what your forerunners think is feasible.

In neutron β-decay about one dozen parameters are accessible by experiment. So far, about half a dozen of these have been measured, some with high precision. As the Standard Model describes neutron decay with only two free parameters, there is ample space for tests beyond the Standard Model. In this conference this state of affairs is mirrored by having altogether four sessions devoted to neutron decay.

At present, one main issue in neutron decay work is the unitarity of Cabibbo-Kobayashi-Maskawa quark-mixing. One would need a 3 sigma shift in the measured neutron β-asymmetry to explain the observed deviation from unitarity, but as much as 10 sigma shifts should any of the other inputs to the analysis be responsible for the deviation. We now have the strange situation that several of the providers of the 10-sigma data claim to be the culprit: the providers of the “strange” matrix element (the most recent paper being arXiv:hep-ph/0307214, 7 May 2004), some of the neutron lifetime providers (this conference), and also the providers of radiative corrections are seen to ponder their heads. The problem, of course, is that many quoted errors are too optimistic. Therefore, when you write in your next funding application that errors will become ten times smaller than those of your competitors, be sure that your students do not feel too much compelled to keep your promises.

In the field of nucleon-nucleon weak interactions, heroic efforts are under way to get hold of parity-violating effects also from simple systems. There weak interaction is used as a tool to derive information orthogonal to the usual nucleon-nucleon strong couplings. In my view, the PNC neutron optics experiments are most beautiful manifestations of parity-violation properties of ordinary matter.

The rich field of neutron wave optics is, as ever, good for surprises. There are new experiments on Bell-inequalities, on non-cyclic and off-diagonal Berry phases, and on the observation of neutron quantum states in the Earth’s gravitational field. This last topic promises to open up a new and rich field, which is very timely as it may lead to new insight into deep current problems of particle physics and cosmology.

Traditionally, a very important point of a conference like this is new methods and instruments for neutron experimentation and, most important, the development of new sources. In past years, we have seen tremendous progress in neutron devices, from neutron guides and polarizers to sophisticated detector systems and others. Physics both drives and is driven by progress in instrumentation. In the field of neutron sources, Japan and the United States will soon have impressive new sources delivering the highest peak fluxes in the world, while Europe will, for some time, continue to have the strongest continuous sources. I hope that the neutron communities will take this as an incentive to exchange projects and people both ways. The fact that several new large neutron source projects are nearing completion also explains the high number of progress reports, so we can expect that our next meeting will give us another explosion of new results.

I shall stop my discussion at this point to let the reader go through the roughly one hundred interesting articles preceding this summary to form his own judgement. Table 1 lists some of the topics covered in the field of neutron-particle physics.

Finally, I want to thank the organizers of this beautiful conference for their work done for our community.

## Figures and Tables

**Table 1:** Sample of questions pursued in neutron-particle physics experiments

Observable	Questions pursued
Neutron electric dipole moment	Why did so much matter survive the Big Bang?
Neutron-antineutron oscillations	Is baryon number conserved?
Neutrino oscillations	Is lepton number conserved?
All of the above:	Are there new symmetries beyond the Standard Model?
Neutron lifetime	What is the number of light neutrino species in the universe? What is the baryon density of the universe? Efficiency of neutrino detectors
Neutron decay correlations	The role of axial coupling in particle physics. Are weak interactions exclusively of the vector-axial vector type? Is ordinary magnetism the *z*-component of electroweak-magnetism? Why do some basic interactions violate time reversal invariance?
Both of the above:	How hot does the sun burn? Is left-right asymmetry an “emergent property” of Nature? Is quark-mixing a “zero-sum game”?
Rare neutron decay modes	How many photons does a neutron beam emit? Is neutron-decay into a hydrogen atom a key to left-right symmetry?
Neutron charge	Why is neutron charge fine-tuned to zero in the Standard Model?
Neutron-neutron strong interactions	Does the *n-n* strong interaction equal the *n-p*, *p-p* strong interaction?
Neutron- nuclear weak interaction	What are the effective nucleon-nucleon couplings?
Neutron-electron scattering length	What is the sign of the neutron squared charge radius?
Neutron electric polarizibility	How steep is the quark-confinement potential?
*ħ/m*_n_, *m*_n_/*m*_p_	What is the strength of the electromagnetic interaction?
Neutron gravity	Does neutron’s inertial mass equal its gravitational mass? Do neutrons fall in quantum steps? Is Newton’s Law valid at small distances/in the quantum regime? Are there compactified extra dimensions of space?
Neutron quantum physics	Spinor 4π rotation/Spin superposition/Squeezed states Topological effects (Aharonov-Casher/Berry) Bell inequality/Dressed neutrons From classical to quantum vibrations/ Linearity of Schrödinger equation/time optics vs space optics, etc.

